# Supplementary material for: Local variations in L/M ratio influence the detection and color naming of small spots
Source: J Vis. 2025 Oct 7;25(12):13. doi: 10.1167/jov.25.12.13 (PMC12514989; doi:10.1167/jov.25.12.13)
Supplement: Supplement 1 [file jovi-25-12-13_s001.pdf]

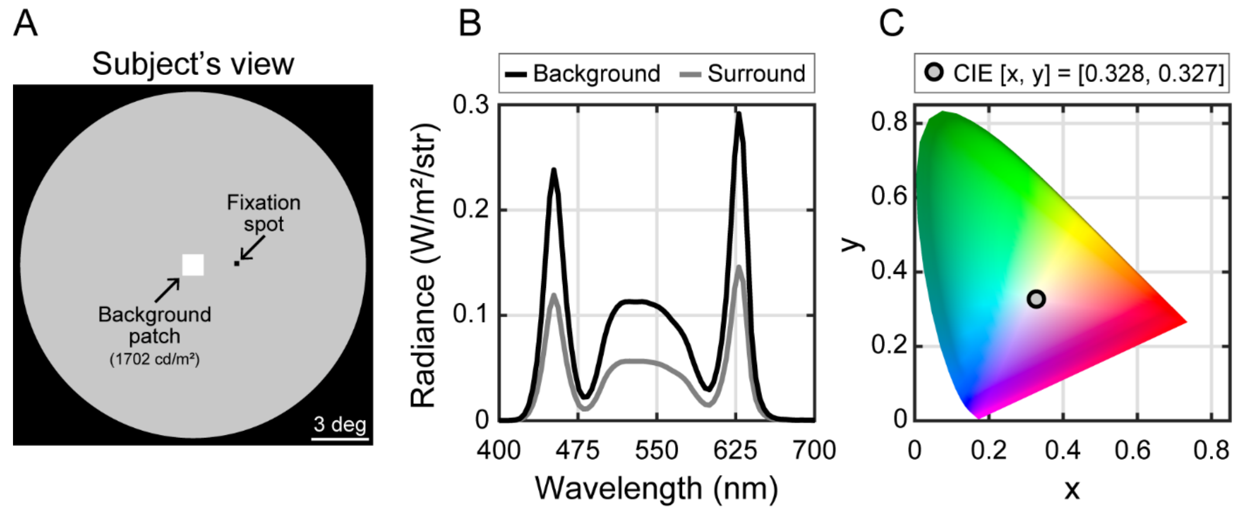

Supplementary Figure 1 | Background configuration and spectral characteristics. (**A**) Diagram depicting the subject's view during the experiment. A 1.1° square white background (1702 cd/m^2) was displayed via a DLP projector configured in Maxwellian view to render the AOSLO imaging raster invisible. The background patch was surrounded by a larger achromatic surrounding field (851 cd/m^2) subtending ~18°. Subject fixation was guided by an adjustable target to ensure the AOSLO raster was aligned to the subject's classified cone mosaic. (**B**) Spectral power distributions of the background (black line) and surround (gray line). (**C**) The chromaticity of the background and surround is plotted in the CIE 1931 *xy* chromaticity diagram.
